# Insights Into the Cultivable Bacterial Fraction of Sediments From the Red Sea Mangroves and Physiological, Chemotaxonomic, and Genomic Characterization of *Mangrovibacillus cuniculi* gen. nov., sp. nov., a Novel Member of the *Bacillaceae* Family

**DOI:** 10.3389/fmicb.2022.777986

**Published:** 2022-02-18

**Authors:** Fatmah O. Sefrji, Ramona Marasco, Grégoire Michoud, Kholoud A. Seferji, Giuseppe Merlino, Daniele Daffonchio

**Affiliations:** Red Sea Research Center (RSRC), King Abdullah University of Science and Technology (KAUST), Thuwal, Saudi Arabia

**Keywords:** *Mangrovibacillus*, mangrove sediments, *Bacillaceae*, cultivable bacteria, diffusion chamber, environmental adaptation, oligotrophic environment

## Abstract

Mangrove forests are dynamic and productive ecosystems rich in microbial diversity; it has been estimated that microbial cells in the mangrove sediments constitute up to 91% of the total living biomass of these ecosystems. Despite in this ecosystem many of the ecological functions and services are supported and/or carried out by microorganisms (e.g., nutrient cycling and eukaryotic-host adaptation), their diversity and function are overlooked and poorly explored, especially for the oligotrophic mangrove of the Red Sea coast. Here, we investigated the cultivable fraction of bacteria associated with the sediments of Saudi Arabian Red Sea mangrove forest by applying the diffusion-chamber-based approach in combination with oligotrophic medium and long incubation time to allow the growth of bacteria in their natural environment. Cultivation resulted in the isolation of numerous representatives of *Isoptericola* (*n* = 51) and *Marinobacter* (*n* = 38), along with several less abundant and poorly study taxa (*n* = 25) distributed across ten genera. Within the latest group, we isolated R1DC41^T^, a novel member of the *Bacillaceae* family in the Firmicutes phylum. It showed 16S rRNA gene similarity of 94.59–97.36% with closest relatives of *Rossellomorea* (which was formerly in the *Bacillus* genus), *Domibacillus*, *Bacillus*, and *Jeotgalibacillus* genera. Based on the multilocus sequence analysis (MLSA), R1DC41^T^ strain formed a separated branch from the listed genera, representing a novel species of a new genus for which the name *Mangrovibacillus cuniculi* gen. nov., sp. nov. is proposed. Genomic, morphological, and physiological characterizations revealed that R1DC41^T^ is an aerobic, Gram-stain-variable, rod-shaped, non-motile, endospore-forming bacterium. A reduced genome and the presence of numerous transporters used to import the components necessary for its growth and resistance to the stresses imposed by the oligotrophic and salty mangrove sediments make R1DC41^T^ extremely adapted to its environment of origin and to the competitive conditions present within.

## Introduction

The number of prokaryotes’ colonies recovered from natural environments and growing on Petri dishes rarely match direct cell counts. Appreciable differences between the two have been observed in many studies (Jannasch and Jones, 1959; Barer and Harwood, 1999), leading to the recognition of the “great plate count anomaly” (Staley and Konopka, 1985). In the last decade, the widespread use of culture-independent molecular methods, such as metagenomics, has confirmed that the majority of the microbial diversity present in the environment are difficult or impossible to culture (Rappé and Giovannoni, 2003; Solden et al., 2016; Dance, 2020). Because of the importance of microorganisms in ecosystems and metaorganisms functionality (Bang et al., 2018; Soldan et al., 2019; Chen et al., 2020; Delgado-Baquerizo et al., 2020; Zamkovaya et al., 2021), the possibility to cultivate microbial “dark matter” is fundamental to increasing our knowledge regarding the dynamics of natural systems and also identify novel genes and their products that are potentially valuable in biological and biotechnological applications, such as useful enzymes, new antimicrobial agents, and other secondary metabolites (Panizzon et al., 2015; Chen et al., 2020; Dance, 2020).

Here, we used the Red Sea mangrove forest as model ecosystem. Mangrove forests are dynamic (Kristensen et al., 2012; Hossain and Nuruddin, 2016; Booth et al., 2019b) and productive ecosystems (Feller et al., 2010) rich in microbial diversity with both high number of bacterial cells and a large number of taxa (Sheaves, 2009; Donato et al., 2011; Imchen et al., 2017; Santana et al., 2019), representing an important untapped source of novel yet uncultured microorganisms. It has been estimated that in mangrove sediments, microorganisms (i.e., bacteria and fungi) constitute 91% of the total biomass (Pramanik et al., 2019), with 10^9^–10^11^ cells per gram of sediment (Alongi, 2013). However, microorganisms in this ecosystem experience intense environmental selection mediated by both abiotic (e.g., nutrient availability, and fluctuations in salinity, temperature, and oxygen concentration) and biotic factors (e.g., animal and plant bioturbation) that can affect the overall biogeochemical nature of the sediments and consequently the microbial diversity harbored (Hossain and Nuruddin, 2016; Booth et al., 2019a,b). In particular, since the Red Sea does not receive freshwater inputs by major rivers or rainfalls and the water circulation through the Suez Chanel is limited (Raitsos et al., 2013), the salinity of its coastline sediments can reach up to 15% during dry summers (Booth et al., 2019b). Moreover, it has been showed that the prevalent biogenic coastal habitats of this sea (i.e., seagrasses, coral reef and mangroves) can experience maximum temperature of 39.2°C (Giomi et al., 2019), making such habitats experimental laboratories for research and discover neglected microorganisms selected by unique extreme environmental conditions.

While, more than 100 microbial species from mangroves ecosystems have been described, only four were obtained from Red Sea sediments (Lee et al., 2012; Zheng et al., 2015; Sefrji et al., 2021a,b), including two from mangroves (Lee et al., 2012; Zheng et al., 2015; Sefrji et al., 2021a,b). Using a diffusion chamber (DC) system (Kaeberlein et al., 2002), we reproduced the chemical milieu of the Red Sea mangrove sediments to facilitate the cultivation and isolation of new bacteria in laboratory conditions.

## Materials and Methods

### Isolation and Habitat

Sampling was performed at the Ibn Sina Field Research Station and Nature Conservation Area (22.34°N, 39.09°E) of the King Abdullah University of Science and Technology (KAUST), Saudi Arabia. Mangrove sediments (Supplementary Figure 1a) were sampled using a sterile spoon and then used as bacterial inoculum for the cultivation experiments. Samples of mangroves’ dead leaves from the same area were also collected (Supplementary Figure 1b) to be used as supplemental nutrients in the preparation of the growth medium. Preparation, inoculation and incubation of diffusion chamber (DC) were performed following the protocol described by Sefrji et al. (2021a). Fresh sea water (FSW) collected from Red Sea was used as medium; for FSW composition refer to Merlino et al. (2018). After 21 days of incubation at room temperature (range, 23–25°C), the DCs were taken out from the aquarium containing mangrove sediments and filtered sea water, washed in pure MilliQ water, and opened under a laminar flow hood. FSW-agar within DC was homogenized and diluted with fresh FSW-agar and leaf extracts to obtain final solutions of 10^–4^ g ml^–1^; these solutions were poured into Petri dishes and further incubated at 26°C for 7 days. By using glass Pasteur pipettes (Sigma-Aldrich) bacterial colonies were picked up, transferred onto agar plates prepared with Luria–Bertani (LB, Difco) diluted 10-time (2 g/L) in FSW and supplemented with 0.1% (vol/vol) of mangrove leaf extract and incubated at 26°C. All plates were incubated for 14 days. Bacteria grown on plates were further selected based on their different morphology (*n* = 150) and transferred and purified following subsequential cultivation on the same medium at 26°C; a total of 116 bacteria were finally retrieved for the further purification steps and analyses. The obtained bacteria were further cultivated in Marine Broth (MB; 5 g peptone, 1 g yeast extract, 0.1 g C_6_H_5_FeO_7_, 19.45 g NaCl, 5.9 g MgCl_2_ • 6H_2_O, 3.24 g MgSO_4_ • 4H_2_O, 1.8 g CaCl_2_, 0.55 g KCl, 0.16 g NaHCO_3_, 0.08 g KBr, 34 mg SrCl_2_ • 6H_2_O, 22 mg H_3_BO_3_, 4 mg Na_2_SiO_3_, 2.4 mg NaF, 1.6 mg NH_4_NO_3_, 8 mg Na_2_HPO_4_). Bacterial culture stocks were prepared in MB containing 30% glycerol and were stored at −80°C

### DNA Extraction and Phylogenetic Analysis of Bacterial Isolates

A total of 116 bacterial strains were obtained and their genomic DNA was extracted by boiling in 50 μl of 10 mM sterile Tris–HCl buffer (pH 8.0) following the protocol described by Marasco et al. (2012). Using universal primer sets that amplify three partially overlapping regions of the bacterial 16S rRNA gene (27F/785R, 341F/907R and 785F/1492R) were used following the protocol previously described (Sefrji et al., 2021a). PCR products obtained were sequenced via Sanger sequencing at the Bioscience Core Lab (KAUST). Electropherograms of the sequences were checked for quality, edited, and assembled using Geneious v. 8.1.9 (Biomatters) to obtain almost full-length sequences (range, 1,300 and 1,450 bp) of the bacterial 16S rRNA gene. The sequences obtained were finally aligned against the National Centre for Biotechnology Information (NCBI) database by using the Basic Local Alignment Search Tool (blast) algorithm in order to phylogenetic identify the closest relative of the isolated bacteria. The sequences were submitted to NCBI under the accession numbers MZ569723–MZ569835. Enterobacterial repetitive intergenic consensus (ERIC)-PCR (De Bruijn, 1992) was performed to examine the clonality of three isolates with the same 16S rRNA gene sequence. ERIC-PCR products are visualized by bioanalyzer Agilent 2100, using high sensitivity dsDNA kit.

### Genome Sequencing and Analyses

The genomic DNA of strain R1DC41^T^ was extracted from 1 ml of liquid culture corresponding to approximately 2 × 10^9^ cells/ml using the Maxwell RSC Automated Nucleic Acid Purification system and the Maxwell^®^ RSC Cultured Cells DNA kit (Promega). Bacterial cultures were grown at 26°C in MB medium for 48 h. DNA concentration was quantified using the Qubit^®^ dsDNA assay with the broad range sensitivity kit (Thermo-Fisher Scientific). The quality was assessed by electrophoresis on 1% agarose gels and using Bioanalyzer 2100 (Agilent). Sequencing was performed at the KAUST Bioscience Core Lab using one cell of the PacBio RS2 platform (Pacific Biosciences). Next, the high quality reads were assembled using the SMRT analysis software (Ono et al., 2013) and hierarchical genome-assembly process.3 workflow (Chin et al., 2013). First, the longest reads were selected as “seed” reads, to which all other reads were mapped. Second, these reads were pre-assembled into highly accurate reads and used for genome assembly. Finally, using all the reads, a final assembly step was performed to correct the initial assembly. The CheckM software was used to determine the presence of contamination in the genome (Parks et al., 2015). The automated annotation pipeline for microbial genomes PROKKA v 1.12 (Seemann, 2014) was used to predict the genes within the contig. Based on the complete 16S rRNA gene sequence from the draft genome of strain R1DC41^T^, the CLUSTALW algorithm within the MEGA software v 10.1.8 (Kumar et al., 2016) was used for sequence alignments and the phylogenetic analysis of the 16S rRNA gene sequence of strain R1DC41^T^ and phylogenetically related taxa obtained from the NCBI database. A phylogenetic tree was constructed using the neighbor-joining and maximum likelihood methods in the MEGA software. The stability of relationships was assessed using a bootstrap analysis based on 1,000 resamplings. Another phylogenetic tree of 120 concatenated single-copy genes, obtained from the genome of strain R1DC41^T^ and compared with > 23,000 bacterial genomes from the Genome Taxonomy Database,^1^ was constructed based on multilocus sequence analysis (MLSA) using the GTDB-Tk software (Parks et al., 2018; Chaumeil et al., 2019). A bootstrap analysis of 1,000 resamplings was used to evaluate the tree topology (Felsenstein, 1985). The genome sequence of the isolate of interest and those of closely related species were imported into the JSpeciesWS, an online service for *in silico* calculation of the extent of identity between genomes. The online software measures the average nucleotide identity (ANI) based on BLAST (ANIb). JSpeciesWS indicates whether two genomes share genomic identity above or below the species embracing thresholds (Burall et al., 2016; Richter et al., 2016). Furthermore, digital DNA–DNA hybridization (DDH) and, Average Amino Acid Identity (AAI) were, respectively, calculated using the GGDC software and the AAI calculator (Richter and Rosselló-Móra, 2009; Rodriguez-R and Konstantinidis, 2016), while the percentage of conserved proteins (POCP) was calculated using the method described by Qin et al. (2014). Additional annotation and gene functional prediction analysis was achieved using the Kyoto Encyclopedia of Genes and Genomes (Kanehisa et al., 2017) via the KofamScan software (Aramaki et al., 2020) and the MagicLamp Software (Garber, 2020). The PHASTER software (Arndt et al., 2016) was used to predict the presence of phages in the genome. The 16S rRNA gene sequence and whole genome were uploaded to GenBank under the accession numbers MT146882 and CP049742, respectively.

### Morphological, Physiological, and Chemotaxonomic Characterization

The recommended minimal standards for describing new taxa of aerobic endospore-forming bacteria were followed (Logan et al., 2009). Cell morphology and the presence of endospores were examined under a scanning electron microscope (Teneo SEM-FEI) at the Imaging Core Lab of KAUST. The standard Gram-staining protocol was used to evaluate the physical properties of bacterial cell wall (Madigan et al., 2008). Swarming motility was assessed in semi-solid MB containing 0.3% agar. The ability of R1DC41^T^ to grow at 10, 20, 30, 40, 45, and 50°C was assessed by inoculating the strain in flask with 50 ml of MB medium, while its growth under different salt concentrations was tested adding to the saltless-MB (i.e., prepared without NaCl, MgCl_2_ • 6H_2_O, Na_2_SO_4_, CaCl_2_, and KCl salts) different amounts of NaCl (range, 0–19%, with serial increments of 1%). Every 24 h, the OD_600_ was measured using the UV-1600PC Spectrophotometer (VWR). Other phenotypical characterizations were performed using the Phenotype Microarray (PM) Biolog^®^ technology that employs the irreversible reduction of tetrazolium violet to formazan as an indicator of active metabolism. R1DC41^T^ was tested for its capacity to survive in the presence of 96 ions/osmolytes (PM9), and different pH (range, 3.5–10; PM10; wells, A1–A12). As well, we evaluate the capacity of R1DC41^T^ strain to use 190 carbon sources within PM1 and PM2. The preparation of media, inoculation, and incubation of PM plates were performed according to the manufacturers’ instructions. During the incubation, the bacterial respiration/metabolism activates the reduction of NAD^+^ into NADH. The NADH produced further acts as reductant of the tetrazolium dye (yellow color) added in the PM medium; this reduced form of the tetrazolium dye, formazan, assumes a blue/purple color that can be quantified and used as indicator of the bacterial metabolism; i.e., more the color is intense, more the bacterial metabolism/respiration is active. The oxidase activity of the R1DC41^T^ strain was tested using oxidase test strips (Sigma-Aldrich), and the catalase activity was tested using 3% hydrogen peroxide solution. Indole production was evaluated by adding 500 μl of Kovacs’ reagent (Sigma) to bacterial culture grown 3 days in MB and tryptophan. The nitrate reduction ability was determined using the nitrate reduction kits (Sigma) following manufacturers’ instructions. The nitrate broth medium was modified and supplemented with 4% NaCl. Amylase, protease, lipase, and cellulase activities were evaluated by streaking R1DC41^T^ on MB agar plates containing 1.5% starch, casein, tween 80, and cellulose, respectively, as substrates, following the instructions described by Liu et al. (2019). At the laboratory of Identification Service, DSMZ (German Collection of Microorganisms and Cell Cultures GmbH, Braunschweig, Germany) the bacterial cells of R1DC41^T^, *Rossellomorea aquimaris* TF-12^T^, *Rossellomorea marisflavic* TF-11^T^; *Jeotgalibacillus soli* P9^T^, *Domibacillus iocasae* S6^T^ were cultured in MB medium under the same conditions to analyze fatty acids, polar lipids, and respiratory quinones.

## Results and Discussion

### Diversity of the Cultured Bacterial Fraction of Mangrove Sediments

**FIGURE 1:**
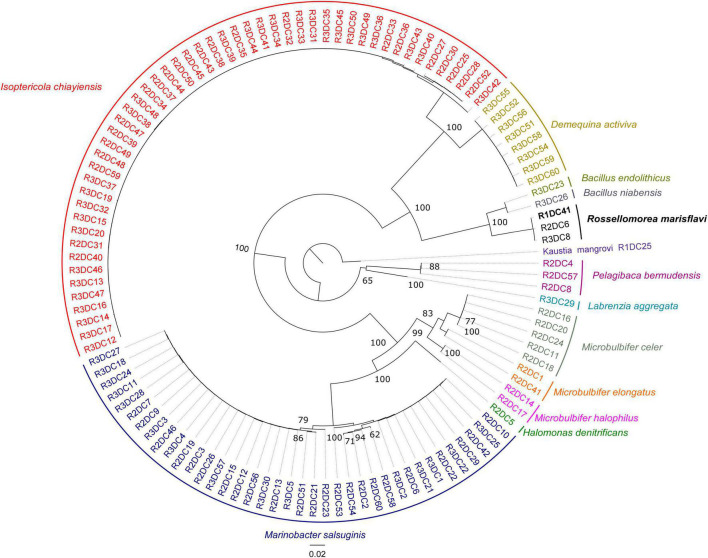
Diversity of cultivable bacteria from mangrove sediments. Neighbor-joining phylogenetic tree based on the 16S rRNA gene sequences and reconstructed using the MEGA X software. Only bootstrap values (expressed as percentages of 1,000 replications) of > 50% are shown at branching points; the colors on the outer rings refer to the bacterial species and to the abundance of that bacteria in our collection (total, *n* = 115), respectively.

The taxonomic identification through the analysis of the bacterial 16S rRNA gene amplified from 116 isolates revealed 13 unique bacterial phylotype (sequencing similarity, 99%). These strains belong to 9 different genera and 13 different species (Figure 1 and Supplementary Table 1). The cultivation strategy applied allowed the isolation of numerous representatives of previously described genera from mangrove sediments, such as *Isoptericola* (*n* = 51), *Marinobacter* (38), along with several less abundant genera, including *Microbulbifer* (9), *Demequina* (8), *Pelagibaca* (3), *Bacillus* (2), *Halomonas* (1), and *Rossellomorea* (3). Among the isolates we detected three strain, namey R1DC41^T^, R2DC6 and R3DC8, that showed 100% identity 16S rRNA gene sequences (Supplementary Figure 2a), the same ERIC-PCR pattern (Supplementary Figure 2b) and similarity values of 97.36% with *Rossellomorea marisflavi* (Figure 1 and Supplementary Table 1). Notably, by using a published dataset that analyzed the microbial community composition of sediment in the same mangrove forest (Booth et al., 2019b), we found that sequences ascribable to the 16S rRNA gene of the three isolates account for a limited portion of reads in the bacterial community (average relative abundance, 0.01%), suggesting their belonging to the rare microbial biosphere (Pedrós-Alió, 2012). The low abundance of colonies observed during the cultivation is compatible with the prevalence of this bacterium within the sediment microbiome observed from the 16S rRNA gene amplicon libraries. As well, such observation confirms previous studies that have shown how members of the rare biosphere can be selected during *in vitro* cultivation (Shade et al., 2012; Crespo et al., 2016).

**FIGURE 2:**
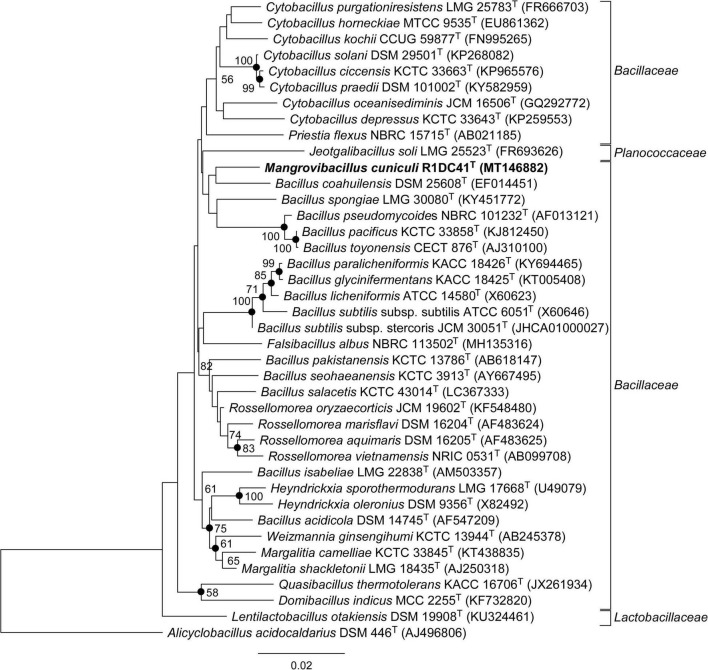
Neighbor-joining phylogenetic tree based on 16S rRNA gene sequences showing the position of R1DC41^T^ strain (MT146882) and other closely related members based on 16S rRNA gene sequences and reconstructed using the MEGA X software. Only bootstrap values (expressed as percentages of 1,000 replications) of > 50% are shown at branching points. Filled circles indicate branches that were also recovered using the maximum likelihood method. *Alicyclobacillus acidocaldarius* DSM 446^T^ (AJ496806) was used as the outgroup. Families of the different strains are found on the right of the phylogenetic trees; bar, 0.02 nt substitutions per nucleotide position.

**FIGURE 3:**
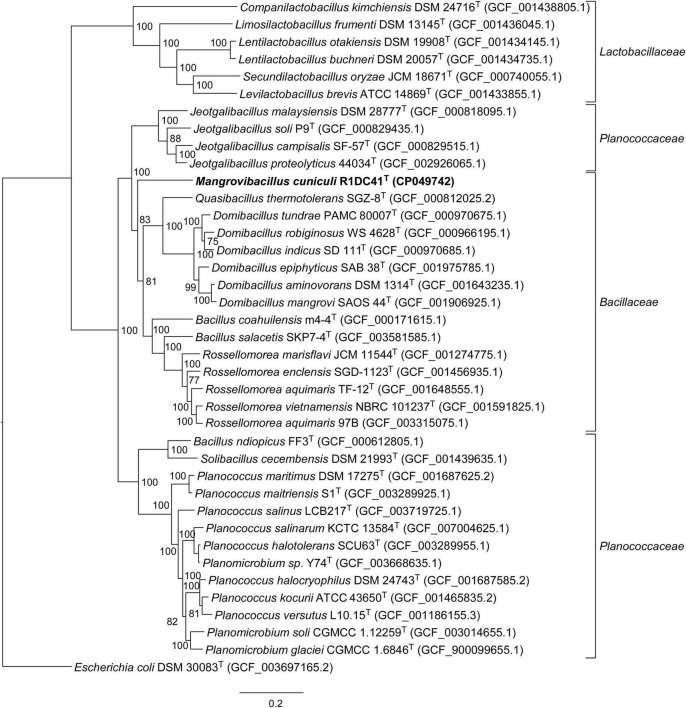
MLSA phylogenetic tree, based on the neighbor-joining algorithm, of R1DC41^T^ strain, and other closely related members of the family *Bacillaceae*, *Lactobacillaceae*, and *Planococcaceae* based on 120 concatenated single-copy genes reconstructed using the GTDB-Tk software. Bootstrap values based on 1,000 replications are listed as percentages at branch points. Bar, 0.2 nt substitutions per nucleotide position.

We selected one of the three isolates, named R1DC41^T^, for genome sequencing and further phylogenetic and physicochemical characterization. Phylogenetic analysis of the 16S rRNA genes (100% similarity between R1DC41^T^ sequences from amplicon PCR and genome) showed sequence similarity values of 97.36 and 97.22% with the closest relatives *Rossellomorea marisflavi* TF-11^T^ and *Rossellomorea aquimaris* TF-12^T^, respectively (Table 1); lower sequence similarities were found between strain R1DC41^T^ and the different type species of the genera *Jeotgalibacillus* (94.18–94.96%; 8 species analyzed), *Quasibacillus* (94.73%), and *Domibacillus* (93.10–94.59%; 9 species analyzed; refer to Supplementary Table 2). Comparative phylogenetic analysis based on the 16S rRNA gene confirmed that the strain formed a distinct clade within the *Bacillaceae* family but it cannot be clearly differentiated from others *Bacillus, Cytobacillus, Priestia*, and *Jeotgalibacillus* species due to the high similarity of the 16S rRNA gene between members of these genera (Gupta et al., 2020) that resulted in low bootstrap support for the phylogenetic tree (Figure 2). However, the use of MLSA based on 120 concatenated single-copy genes of bacterial genomes indicated a distinct position for R1DC41^T^ with respect to the other closely related species within the *Bacillaceae* families, providing additional evidence for the phylogenetic placement of our strain as a novel genus within this family (Figure 3). We also note that in a recent paper (Gupta and Patel, 2020) the taxonomy of both *Bacillus ndiopicus* FF3^T^ and *Bacillus cecembensis* DSM 21993^T^ was modified to *Metasolibacillus ndiopicus* and *Solibacillus cecembensis* belonging to the *Planococcaceae* family; according to the List of Prokaryotic names with Standing in Nomenclature (LPSN) (Parte et al., 2020), only *Solibacillus cecembensis* was validly published, hence we changed only this name in the phylogenetic tree (Figure 3). Analyses of ANib, DDH, and POCP between strain R1DC41^T^ and closest related species gave values in the range of 66–68%, 23–36%, and 14–48%, respectively (Table 1). Both ANIb and DDH values were below the respective thresholds for new species, 95 and 70%, respectively, and the POCP was below the threshold of 50% for genus delimitation (Meier-Kolthoff et al., 2013; Qin et al., 2014; Richter et al., 2016). Furthermore, the AAI analysis showed how similarity between our strain and all the other closest species range between 40 and 55% (Table 2), values below the threshold for AAI genus delimitation (Konstantinidis et al., 2017). The data obtained from the phylogenetic analyses indicated that R1DC41^T^ (= KCTC 43068^T^ = NCCB 100700^T^ = JCM 33621^T^) represents a novel species of a new genus within the *Bacillaceae* family, Firmicutes phylum, for which the name *Mangrovibacillus cuniculi* gen. nov., sp. nov. is proposed.

**TABLE 1:** Percentage identity of 16S rRNA gene comparisons, Average Nucleotide Identity via BLAST (ANIb), and percentage of aligned sequences in brackets, digital DNA–DNA hybridization (DDH), and percentage of conserved proteins (POCP) matrix of our isolate R1DC41^T^ and other closely related species belonging to the *Bacillaceae*, *Lactobacillaceae*, and *Planococcaceae*; the full list of closest related analyzed for each genus is reported in Supplementary Table 2.

Reference genome	16S rRNA (%)	ANIb (%)	DDH (%)	POCP (%)
*Rossellomorea aquimaris* TF-12^T^	97.36	68.36 (30.65)	27.7	14.35
*Rossellomorea marisflavi* TF-11^T^	97.22	67.66 (31.37)	23.8	39.45
*Bacillus coahuilensis* m4-4^T^	95.93	69.08 (35.47)	35.6	48.35
*Jeotgalibacillus soli* P9^T^	94.96	68.63 (30.94)	36.25	36.79
*Quasibacillus thermotolerans* KACC 16706^T^	94.73	68.34 (26.80)	27.3	44.38
*Domibacillus indicus* MCC 2255^T^	94.59	67.57 (25.04)	25.6	37.5
*Planococcus maritimus* DSM 17275^T^	93.41	67.12 (22.88)	29.15	38.48
*Secundilactobacillus oryzae* JCM 18671^T^	89.51	66.47 (7.53)	25.7	41.97

*The cutoff for species discrimination was ≥ 97% for 16S rRNA gene comparison, ≥ 95% and ≥ 70% for ANIb and DDH, respectively, whereas the cutoff for genus discrimination was ≥ 50% for POCP.*

**TABLE 2:** Average Amino Acid Identity (AAI) of R1DC41^T^ strains in comparison with other closely related species belonging to the *Bacillaceae*, *Lactobacillaceae*, and *Planococcaceae*.

Strain	R1DC41^T^	m4-4^T^	JCM 18671^T^	KACC 16706^T^	P9^T^	MCC 2255^T^	TF-11^T^	TF-12^T^	DSM 17275^T^
R1DC41^T^	100								
m4-4^T^	42.2	100							
JCM 18671^T^	55.7	57.5	100						
KACC 16706^T^	42.4	43.0	54.9	100					
P9^T^	42.1	42.7	55.7	41.2	100				
MCC 2255^T^,	43.7	44.7	55.7	36.4	41.9	100			
TF-11^T^	40.9	40.2	55.5	39.9	40.3	42.3	100		
TF-12^T^	40.0	38.7	55.7	39.1	39.4	41.2	28.2	100	
DSM 17275^T^	45.2	46.5	56.6	43.8	43.0	44.7	44.1	43.9	100

*All values are expressed in percentage (%). The cutoff values for AAI values are < 45%, 45–65% and 65–95% for (different) family, genus and species, respectively. 1: R1DC41^T^, strain of this work; m4-4^T^, Bacillus coahuilensis; JCM 18671^T^, Secundilactobacillus oryzae; KACC 16706^T^, Quasibacillus thermotolerans; P9^T^, Jeotgalibacillus soli; MCC 2255^T^, Domibacillus indicus; TF-11^T^, Rossellomorea marisflavi; TF-12^T^, Rossellomorea aquimaris; DSM 17275^T^, Planococcus maritimus.*

### Phenotypic and Chemotaxonomic Characterization of Novel R1DC41^T^ Strains

**FIGURE 4:**
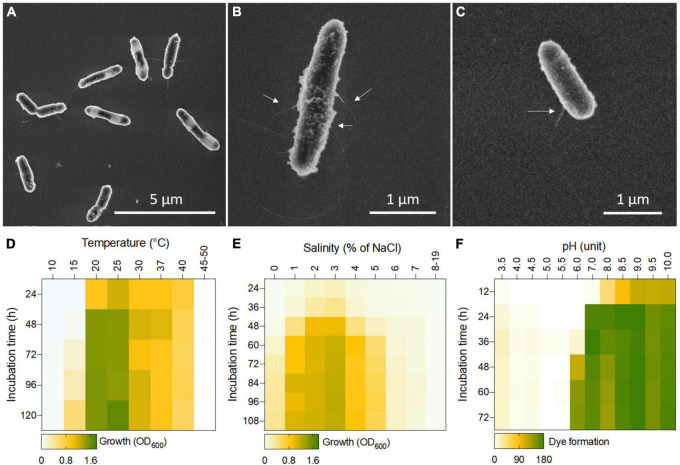
**(A)** Scanning electron micrograph of R1DC41^T^ culture growth in MB at 26°C for 3 days depicts rod-shaped bacteria cells with pili; bar length, 5 μm. **(B,C)** Individual cells from the suspension cultures of R1DC41^T^ show pili (white arrows); at least one pilum per cell is detected. Flagellar structures were not detected. Bar length, 1 μm. **(D–F)** Heat maps showing the growth of R1DC41^T^ at different **(D)** temperatures and **(E)** salinity (% of NaCl) indicated as mean values of OD_600_; **(F)** metabolic activity of R1DC41^T^ under different pH are reported as mean values of blue/purple color formation due to tetrazolium dye reduction.

The bacterial cells of R1DC41^T^ strain form pale-yellow, circular, smooth colonies with irregular edges when grown on MB agar medium (4% salinity) at 26°C. Its cells are Gram-stain-variable (initially Gram-stain-negative and becoming Gram-stain-positive in the later stages of growth), perform aerobic respiration, have a rod shape (0.4–0.6 μm wide and 1.7–2.3 μm long), and produce central or subterminal endospores (Figures 4A–C) as indicated by the presence of the *spo0ABF* genes responsible for sporulation (Jiang et al., 2000) in the genome (Table 3). Such ability provide enhanced resistance to heat, radiation, and drought, facilitating the bacterial survival in the harsh conditions of the Saudi mangrove forest for prolonged periods (Booth et al., 2019b; Anton et al., 2020; Arshad et al., 2020). Despite the presence of several genes involved in flagella synthesis and chemotaxis (Table 3) and the presence of small and rare peritrichous flagella (Figures 4B,C), active flagellum-mediated motility (i.e., swarming) was not observed *in vitro*. Furthermore, no genes involved in pili formation, gliding mobility, or quorum sensing could be identified in the genome. It is common for bacterial strains cultivated and domesticated under laboratory conditions to lose their ability to produce flagellar structures and motility (Mukherjee and Kearns, 2014). In addition, according to the environmental niche of origin of the R1DC41^T^ strain (i.e., mangrove sediment), adhesion to sediment particles mediated by flagella may be sufficient to allow the bacterium to flourish by exploiting the constant replacement of nutrients and carbon sources by the tidal flows, invertebrate bioturbation activity, and mangrove litter (Moens and Vanderleyden, 1996; Booth et al., 2019b).

**TABLE 3:** Genes associated with adaptive traits, transports of metabolites and mobility in *Mangrovibacillus cuniculi* R1DC41^T^.

Function	Gene name	Best hit (G8O30_)
**Adaptive traits**		
Osmoprotectant	*proA, proB, proC, lysC, opuA. opuB, opuC*	05805, 05810, 05815, 09360, 05615, 05620, 05625
Phosphate limitation	*phoA, phoD, phoP, phoR*	05860, 00985, 09565, 13505
Oxygen limitation	*resD, resE CtaA*	07045, 07050, 04200
Temperature stress	*vicK, vicR, desK, desR, Des*	13505, 13510, 10070, 10065, 10075
Antibiotic resistance	*LiaG, liaF, liaI, liaS, liaR*	09665, 11190, 01490, 01480, 01495, 01500
Siderophore synthesis	*menF*	10230
Carotenoid production	*crtB, crtI, crtO, crtP, crtQ*	03230, 03220, 06265, 05935, 05940
Sporulation	*spo0A, spo0B, spo0F*	07485, 08710, 12615
**ABC transporters**		
Galactose	*ganO, ganP, ganQ, msmX*	03255, 03260, 03265, 02795, 02820
Lactose/arabinose	*lacE, lacF, lacG*	00380, 00385, 00390
Nucleoside	*bmpA, nupA, nupB, nupC*	01560, 03345, 05190, 05195. 05205, 05200
Raffinose	*msmE, msmF, msmG, msmK*	01955, 00385, 01945, 01950, 02820
Cystine	*tcyA, tcyB, tcyC*	02785, 02790, 02795
Methionine	*metI, metN, metQ*	11800, 11805, 11810
Oligopeptide	*oppA, oppB, oppC, oppD, oppF*	01775, 03415, 03420, 03425, 01770, 03375, 03430,
Phosphate	*pstA, pstB, pstC, pstS*	07870, 07875, 07880, 07885
Phosphonate	*phnC, phnD, phnE*	03795, 03800, 03805, 02785
Biotin	*bioY, ecfA1, ecfA2, ecfT*	05780, 14515, 14520, 14525
Zinc	*znuA, znuB, znuC*	05630, 07955, 07960
Zinc/Iron/Manganese	*troA, troB, troC, trod*	06520, 06515, 06510, 06505
**Mobility**		
Chemotaxis	*cheA, cheB, cheC, cheD, cheV, cheW, cheX, cheY*	04985, 04980, 04995, 05000, 03920, 04990, 06580, 04935
Flagella	*flgB, flgC, flgD, flgE, flgG, flgK, flgL, flgM, flgN, flhA, flhB, fliA, fliC, fliD, fliE, fliF, fliG, fliG, fliH, fliI, fliJ, fliK, fliL, fliM, fliM, fliN, fliN, fliO, fliP, fliQ, fliR, fliS, fliT*,	04850, 04855, 04900, 04910, 12355, 11430, 11425, 11440, 11435, 04965, 04960, 05010, 11395, 11370, 04860, 04865, 04870, 04875, 04880, 04885, 04895, 04920, 04925, 04930, 04940, 04945, 04950, 04955, 11365, 11360

R1DC41^T^ is a mesophile that can grow between 20 and 40°C, with a growth optimum at 26°C (Figure 4D). It actively grows from a sub-acid pH of 6 to and alkaline pH of 10, with an optimum pH of 8.5–9 (Figure 4E); it can be classified as a moderate alkaliphile, confirming its ability to survive and grow under the alkaline condition of mangrove sediments, which has a pH of 6.5–10. Despite the accumulation of salts in the mangrove ecosystem, especially during the summer periods when water evaporates at a high rate and salts accumulate on the sediment surface (Booth et al., 2019b), R1DC41^T^ is able to grow only between 1 and 5% of NaCl, with a growth optimum of 3–4%; its metabolism is significantly reduced at salinity values above this range (Figure 4F). The reduced bacterial metabolism/respiration at high salt concentration (>5% NaCl) was also confirmed using the Biolog PM9 plate (Supplementary Table 3). These results suggest that salt- and temperature-sensitivity of our strain R1DC41^T^ could be overcome by the production of endospores previously discussed to ensure bacterial survival during the salt accumulation (up to 15%; Booth et al., 2019b) and high temperature (up to 39.2°C; Giomi et al., 2019) occurring in summer. R1DC41^T^ is also able to cope with the osmotic stress induced by other osmolytes and ions, such as potassium chloride, sodium sulfate, sodium phosphate, sodium nitrate, and sodium nitrite (Supplementary Table 3), which are generally present in the Red Sea water and mangrove sediments (Balk et al., 2015; Manasrah et al., 2020). However, the metabolic activity of our strain was drastically inhibited by urea, ammonium sulfate, sodium formate, sodium lactate, sodium benzoate and high concentrations of sodium nitrite (> 20 mM; Supplementary Table 3). In addition, the strain tested negative for the cytochrome c oxidases, but it was positive for the catalase enzyme involved in the protection of the cell from oxidative damage generated by reactive oxygen species (ROS) formed under stress conditions (Nandi et al., 2019).

Strain R1DC41^T^ grows well on gelatin as sole carbon source, and weakly on L-glutamic acid, glycyl-L-glutamic acid, glycyl-L-proline, pectin, oxalomalic acid, and sorbic acid; no growth was detected on the remaining 183 carbon sources available in the Biolog PM1 and PM2 plates. The R1DC41^T^ cells were negative for amylase, protease, lipase, and cellulase enzymes and were unable to reduce nitrate to nitrite or produce indole from tryptophan. These results are in contrast to the lifestyle and ecosystem functions described for the majority of the *Bacillaceae* members (i.e., *Bacillus* and *Rossellomorea*) that are primarily heterotrophic saprophytes capable of degrading a range of polymeric carbonaceous substances present in the soil, such as organic matter and plant litter, and also grow on various simple compounds (Mandic-Mulec et al., 2015).

The major respiratory quinone of strain R1DC41^T^ is menaquinone MK7 (100%), which is the predominant menaquinone in the *Bacillaceae* family members (De Vos et al., 2009). Notably, the closely related *R. aquimaris* (TF-12^T^), *R. marisflavic* (TF-11^T^) and *J. soli* (P9^T^) present also MK8 and/or MK6 and/or MK5, while *D. iocasae* (S6^T^) has as major quinone MK6 (Supplementary Table 5). The fatty acid profile of R1DC41^T^ is dominated by the branched-chain saturated fatty acids iso-C_15:0_, anteiso-C_15:0_, anteiso-C_17:0_, iso-C_16:0_, and iso-C_17:0_ and the straight-chain saturated fatty acid C_16:0_; unsaturated chain fatty acids were present at low levels (Supplementary Table 4). This fatty acid profile of R1DC41^T^ differs from those of the closely related strains, but following different trends when it is compared with *Rossellomorea* strains or *J. soli* or *D. iocasae* (Supplementary Table 4). However, as general trend, we find higher amount of the iso–C_17:0_, along with the absence of C16:1ω7c and summed feature 4 in R1DC41^T^ compared to the other strains analyzed (Supplementary Table 4). These differences support the classification of R1DC41^T^as a new species representative of a novel genus.

The fatty acid profile of strain R1DC41^T^ differs from those described for the phylogenetically close strains of the genera *Rossellomorea*, *Jeotgalibacillus*, *Quasibacillus*, and *Domibacillus*, mainly owing to. Although the cellular polar lipids diphosphatidylglycerol (DPG) and phosphatidylglycerol (PG) were consistently found in strain R1DC41^T^ and its related strains of the *Quasibacillus* and *Domibacillus* genera, two unidentified lipids (L1 and L2) and a minor amount of one unidentified aminolipid (AL1) were also detected (Supplementary Table 5 and Supplementary Figure 3).

Phenotypic, biochemical, and genomic characteristics of strain R1DC41^T^ in comparison with those of the closely related species (i.e., *R. aquimaris*, *R. marisflavi, Jeotgalibacillus soli*, *Quasibacillus thermotolerans*, and *Domibacillus indicus*) are summarized in Supplementary Table 5.

### Genomic Features and Adaptations to Oligotrophic Mangrove Sediments

The size of the genome of strain R1DC41^T^ is 3.2 Mb and G + C content is 38.3 mol%, and the genome can be assembled into one contig (Supplementary Figure 4b). The purity (completeness and contamination) of the genome was confirmed by investigating for the presence of single copy genes via the checkM software (Parks et al., 2015) showing a 99.3% completeness and no contamination (duplicate single copy genes). The total number of predicted genes is 3,304, including 3,151 protein-coding sequences genes (CDS) and 36 ribosomal RNAs. Twelve copies of the 16S rRNA gene (1,574 bp) had 100% similarity with 81 copies of tRNAs. We also predicted the presence of a 43 kb long integrated phage (Supplementary Figure 4b), separated from the genome by the attachment sites *att*L and *att*R (Kim and Landy, 1992). This phage contains 45 CDS, including 12 genes coding for tail, head, capsid, portal, terminase, and integrase proteins along with 23 hypothetical proteins. Other phage genes are involved in DNA replication and transcription, including G8_09135, G8_09215, G8_09220, and G8_09235 that code for DNA topoisomerase II, dsDNA binding protein, recombinational DNA repair protein, and transcription state regulatory protein, respectively. Although some genes (7–9) are homologs to those in already described phages, such as *Geobacillus* phage GBSV1 (NC_008376), *Bacillus* virus 1 (NC_009737), and *Bacillus* phage vB_BhaS-171 (NC_030904), the remaining portion showed low level of similarity (< 30% nucleotide identity) with other described or submitted phages, either integrated or not in bacterial genomes.

Interestingly, we noted the absence of many genes involved in nitrogen and sulfur metabolisms, as well as reduction of those genes involved in carbon utilization with exception from those of core metabolisms, such as glycolysis, TCA cycle, pentose phosphate pathway and oxidative phosphorylation. Most of the amino acids biosynthesis pathways are either absent or incomplete, except cysteine, histidine, proline, and threonine. However, we found many genes encoding ABC transporters involved in the intake of oligosaccharide (galatose, raffinose, lactose/arabinose, cellobiose, nucleoside), amino acids (cystine, methionine), phosphate, phosphonate, oligopeptide, zinc, iron, manganese, and biotin (Table 3). These results suggest that strain R1DC41^T^ streamlined its genome to reduce the number of genes involved in the different metabolisms and thus its energy requirements. While its genome size is 3.2 Mb, those of its closest neighbors ranges from 3.8 to 4.3 Mb (data obtained from genomes; Supplementary Table 5). Indeed, the presence of high microbial biomass in the mangroves, along with its fauna (e.g., crabs, up to 100/m^2^) and vegetations (Alongi, 2013; Andreetta et al., 2014) may allow R1DC41^T^ to obtain the necessary nutrients for its survival and fitness by importing them.

We also noted the presence of many genes involved in the production of two components’ systems related to the adaptation to different stresses (e.g., phosphate and oxygen limitation, sporulation, temperature stress and antibiotic resistance), as well as all genes involved in chemotaxis and flagellar assembly (Table 3). Other genes involved in bacterial adaptation to oligotrophic mangrove sediments (Booth et al., 2019b; Anton et al., 2020) were found, such as those encoding for phosphate solubilization and siderophore synthesis (Table 3). As well, R1DC41^T^ had genes for the production and transport of osmoprotectants, such as proline (*proABC*, G8O30_05805, G8O30_05810, G8O30_05815), ectoine (*lysC*, G8O30_09360), and ABC transporters of glycine betaine *OpuABC* (G8O30_05615, G8O30_05620, G8O30_05625), that suggest its ability to accumulate compatible solutes (e.g., ectoine, glycine betaine, and proline) to withstand the salt and osmotic stresses (Oren, 2011). Beside these adaptations, we detected genes encoding for carotenoid production (Table 3). The presence of genes involved in the production of the intermediate carotenoid lycopene may indicate the presence of catalase activity because lycopene has been shown to contribute to protection against ROS (Bradbury et al., 2012). Furthermore, we detected genes *crtOPQ* (G8O30_05935, G8O30_06265, G8O30_05940) partly responsible for the biosynthesis of staphyloxanthin (Pelz et al., 2005), a carotenoid pigment first described in *Staphylococcus aureus* species; it confer the yellow color to the colonies, as the case for strain R1DC41^T^. This pigment has been shown to have antioxidant action to counteract the effects of ROS and its absence has also been linked to UV sensitivity (Clauditz et al., 2006; Pannu et al., 2019).

### Description of *Mangrovibacillus* gen. nov.

*Mangrovibacillus* (Man.gro.vi.ba.cil’lus. N.L. neut. n. *mangrovum* a mangrove; Gr. n. *baktron* rod; N.L. masc. n. *bacillus* a rod) is a novel bacterial genus from mangrove sediments of the Red Sea. Cells are Gram-stain-variable rods, initially Gram-stain-negative and in the later stages of growth Gram-stain-positive, with central or subterminal endospore formation, aerobic respiration, and moderate halotolerance; cells are also moderate alkaliphilic and mesophilic, are non-motile in semi-solid agar MB medium, and exhibit pili. The major respiratory quinone is MK7, and the major polar lipids are phosphatidylethanolamine, two unidentified phospholipids, and two unidentified lipids. The major cellular fatty acids (> 5%) include the branched-chain saturated fatty acids iso-C_15:0_, anteiso-C_15:0_, anteiso-C_17:0_, iso-C_16:0_, iso-C_17:0_ and the straight-chain saturated fatty acid C_16:0_. The G + C content of the genomic DNA is 38.3 mol%.

### Description of *Mangrovibacillus cuniculi* gen. nov., sp. nov. (cu.ni’cu.li. L. gen. n. *Cuniculi* of a Burrow)

The name “cuniculi” was assigned because the type species was isolated from sediment from a crab burrow in a mangrove forest. In addition to the genus features, this species has the following characteristics. Cells are rod-shaped with a width of 0.4–0.6 μm and length of 1.7–2.3 μm. Colonies are circular, with a diameter of 1–2 mm and regular edges; they are smooth and shiny and have a pale-yellow color when grown on MB agar medium at 26°C. Cells grow at 20–40°C (optimum, 20–25°C) in a medium of pH 6–10 (optimum, pH 8.5–9) and containing 1–5 % NaCl (optimum, 3 –4%). Under optimal conditions (i.e., MB medium at pH 9 and incubation at 26°C), the doubling time for R1DC41^T^ is 3.9 h. Growth with a sole carbon and energy source is mainly supported by gelatin. Catalase activity is positive, whereas oxidase activity is negative; nitrate reduction and indole production from tryptophan are negative. Strain R1DC41^T^ was unable to hydrolyze starch, casein, Tween 80, and cellulose.

The type strain is R1DC41^T^ (= KCTC 43068^T^ = NCCB 100700^T^ = JCM 33621^T^). The GenBank accession numbers for the 16S rRNA gene sequence and the complete genome sequence of strain R1DC41^T^ are MT146882 and CP049742, respectively.

## Data Availability Statement

The datasets presented in this study can be found in online repositories. The names of the repository/repositories and accession number(s) can be found below: https://www.ncbi.nlm.nih.gov/, MZ569723–MZ569835; https://www.ncbi.nlm.nih.gov/genbank/, MT146882 and CP049742.

## Author Contributions

FS, GMe, RM, and DD designed the study. FS, RM, GMi, KAS, and GMe performed the experiments. FS, RM, GMi, and KAS analyzed the data. DD supported the research. RM, GMi, and DD wrote the manuscript with contributions from other authors. All authors contributed to the article and approved the submitted version.

## Conflict of Interest

The authors declare that the research was conducted in the absence of any commercial or financial relationships that could be construed as a potential conflict of interest.

## Publisher’s Note

All claims expressed in this article are solely those of the authors and do not necessarily represent those of their affiliated organizations, or those of the publisher, the editors and the reviewers. Any product that may be evaluated in this article, or claim that may be made by its manufacturer, is not guaranteed or endorsed by the publisher.

## Footnotes

1
http://gtdb.ecogenomic.org
