# Supplementary material for: Insights Into the Cultivable Bacterial Fraction of Sediments From the Red Sea Mangroves and Physiological, Chemotaxonomic, and Genomic Characterization of Mangrovibacillus cuniculi gen. nov., sp. nov., a Novel Member of the Bacillaceae Family
Source: Front Microbiol. 2022 Feb 18;13:777986. doi: 10.3389/fmicb.2022.777986 (PMC8894767; doi:10.3389/fmicb.2022.777986)
Supplement: Supplementary file 1 [file Data_Sheet_1.PDF]

SUPPLEMENTARY MATERIALS

Insights into the cultivable bacterial fraction of sediments from the Red Sea mangroves and physiological, chemotaxonomic, and genomic characterization of *Mangrovibacillus cuniculi* gen. nov., sp. nov., a novel member of the *Bacillaceae* family

Fatmah O. Sefrji[1,#], Ramona Marasco[1,#], Grégoire Michoud[1,#], Kholoud A. Seferji[1], Giuseppe Merlino[1], Daniele Daffonchio[1,*]

[1]Red Sea Research Center (RSRC), King Abdullah University of Science and Technology (KAUST), Thuwal, Saudi Arabia

[#]These authors contributed equally to the work
*Correspondence: Daniele Daffonchio, daniele.daffonchio@kaust.edu.sa

Supplementary Tables

Supplementary Table S1. Closest relatives of the 13 haplotypes identified among the 116 bacterial isolates. For each haplotype is indicated the number of isolates belonging to and the percentage of sequence identity with the closest relative. Star (*) indicate the group of the novel bacteria described in this work, R1DC41^{T}.

N. of isolates	Closest described relative	Identity [%]
3	*Rossellomorea marisflavi*	97.36*
1	*Bacillus endolithicus*	98.92
1	*Bacillus niabensis*	98.88
8	*Demequina activiva*	99.24
1	*Halomonas denitrificans*	99.52
51	*Isoptericola chiayiensis*	99.93
1	*Labrenzia aggregata*	99
4	*Marinobacter adhaerens*	99.31
34	*Marinobacter salsuginis*	100
5	*Microbulbifer celer*	99
2	*Microbulbifer elongatus*	98.98
2	*Microbulbifer halophilus*	98.34
3	*Pelagibaca bermudensis*	99.79

Supplementary Table S2. Percentage of identity obtained by comparing the 16S rRNA genes of R1DC41^{T} with other closely reference strains belonging to *Domibacillus* and *Joetgalibacillus* genera.

Reference	Query cover	e-value	Percentage of identity	Accession
Domibacillus antri XD80^{T}	95%	0	93.31%	NR_149777.1
Domibacillus enclensis NIO-1016^{T}	95%	0	93.39%	NR_134021.1
Domibacillus epiphyticus SAB 38^{T}	92%	0	93.56%	NR_157744.1
Domibacillus indicus SD111^{T}	91%	0	94.59%	NR_134022.1
Domibacillus iocasae S6^{T}	91%	0	93.10%	NR_148625.1
Domibacillus mangrovi SAOS 44^{T}	94%	0	93.48%	NR_157743.1
Domibacillus robiginosus WS 4628^{T}	95%	0	93.68%	NR_108861.1
Domibacillus tundrae PAMC 80007^{T}	95%	0	93.15%	NR_137368.1
Jeotgalibacillus alimentarius YKJ-13^{T}	97%	0	94.18%	NR_114573.1
Jeotgalibacillus alkaliphilus JC303^{T}	90%	0	94.38%	NR_153709.1
Jeotgalibacillus campisalis SF-57^{T}	97%	0	94.84%	NR_025716.1
Jeotgalibacillus malaysiensis D5^{T}	99%	0	94.46%	NR_136485.1
Jeotgalibacillus marinus 581^{T}	98%	0	94.64%	NR_025351.1
Jeotgalibacillus marinus ATCC 29841^{T}	97%	0	94.67%	NR_112057.1
Jeotgalibacillus salarius ASL-1^{T}	97%	0	94.58%	NR_116485.1
Jeotgalibacillus soli P9^{T}	98%	0	94.96%	NR_125726.1
Jeotgalibacillus terrae JSM 081008^{T}	93%	0	94.05%	NR_116713.1

Supplementary Table S3. Metabolic profiling of R1DC41^{T} strain tested in the presence of ions and osmolytes using the Biolog PM9 plate. Active growth (*i.e.*, NADH reduction) was indicated as positive (++), weakly positive (+), or negative (−). In case a molecule is present at different concentration, the range in which the strain can grow (+ and ++) or not (−) is reported.

**Substrate**	**NADH reduction**	**Substrate**	**NADH reduction**
NaCl 1%–5.5%	++	NaCl 6% + Glycerol	+
NaCl 6%–7%	+	NaCl 6% + Trehalose	+
NaCl 8%–10%	−	NaCl 6% + Trimethylamine-N-oxide	+
NaCl 6% + Betaine	+	NaCl 6% + Trimethylamine	+
NaCl 6% + N-N Dimethyl glycine	+	NaCl 6% + Octopine	+
NaCl 6% + Sarcosine	+	NaCl 6% + Trigonelline	+
NaCl 6% + Dimethyl sulphonyl propionate	+	Potassium chloride 3%–6%	++
NaCl 6% + MOPS	+	Sodium sulfate 2%–4%	++
NaCl 6% + Ectoine	+	Sodium sulfate 5%	+
NaCl 6% + Choline	+	Ethylene glycol 5%–20%	+
NaCl 6% + Phosphoryl choline	+	Sodium formate 1%–6%	−
NaCl 6% + Creatine	+	Urea 2%–7%	−
NaCl 6% + Creatinine	+	Sodium Lactate 1%–12%	−
NaCl 6% + L-Carnitine	+	Sodium Phosphate pH 7 20–200 mM	++
NaCl 6% + KCl	+	Sodium Benzoate pH 5.2 20–200 mM	−
NaCl 6% + L-proline	+	Ammonium sulfate pH 8 10–100 mM	−
NaCl 6% + N-Acethyl L-glutamine	+	Sodium Nitrate 10–40 mM	++
NaCl 6% + ß-Glutamic acid	+	Sodium Nitrate 60–100 mM	+
NaCl 6% + γ-Amino-n-butyric acid	+	Sodium Nitrite 10 mM	++
NaCl 6% + Glutathione	+	Sodium Nitrite 20–100 mM	−

Supplementary Table S4. Cellular fatty acid composition (%) of R1DC41^T strain and closely related members within the *Bacillaceae* family. Bacteria have been grown on MB medium and analysed were carried out by DSMZ Services, Lebniz-Institut DSMZ.

**Fatty acid type**	**Fatty acid**	**R1DC41^T**	**TF-12^T**	**TF-11^T**	**P9^T**	**S6^T**
**Cultural medium**		Marine agar	Marine agar	Marine agar	Marine agar	Marine agar
**Saturated straight-chain**	$C_{14:0}$	1.96	TR	TR	TR	**13.62**
	$C_{16:0}$	**5.61**	TR	TR	TR	**8.29**
	$C_{18:0}$	–	–	–	TR	TR
**Saturated branched-chain**	iso–$C_{14:0}$	1.49	**5.26**	**10.78**	1.14	1.99
	anteiso–$C_{15:0}$	**12.0**	**14.80**	**29.18**	**52.60**	**11.96**
	iso–$C_{15:0}$	**52.05**	**52.42**	**23.93**	**17.86**	**17.60**
	iso–$C_{16:0}$	**7.57**	2.56	**11.49**	TR	3.03
	iso–$C_{17:0}$	**6.65**	TR	1.64	2.23	1.40
	anteiso–$C_{17:0}$	**9.60**	1.21	**10.03**	**7.58**	2.44
**Unsaturated branched-chain**	iso–$C_{16:1}$ H	–	–	–	–	1.32
	Iso-$C_{15:1}\omega 9c$	–	1.52	–	TR	–
	$C_{16:1}\omega 7c$	–	**10.05**	**5.41**	**2.85**	–
	$C_{16:1}\omega 11c$	TR	1.90	–	4.07	**17.67**
	iso–$C_{17:1}\omega 10c$	1.0	4.36	–	1.96	–
**Hydroxy**	Iso–$C_{14:0}$ 3–OH	–	TR	TR	TR	–
	Iso–$C_{15:0}$ 3–OH	–	–	TR	–	–
	$C_{15:0}$ 2–OH	–	–	TR	–	–
	Iso–$C_{16:0}$ 3–OH	–	–	TR	–	–
**SF3***		TR	–	–	–	**12.66**
**SF4***		–	3.75	3.15	**7.31**	2.36

R1DC41^T *Mangrovibacillus cuniculi* (this study); TF-12^T *Rossellomorea aquimaris* [1]; TF-11^T *Rossellomorea marisflavic*[1]; P9^T *Jeotgalibacillus soli* [2]; S6^T *Domibacillus iocasae* [3].
*: summed features (SF) are groups of two or three fatty acids that could not be separated via GLC using the MIDI system; SF3 comprises iso-$C_{15:0}$ 2-OH and/or $C_{16:1}\omega 7c$ and/or $C_{16:1}\omega 6c$, whereas SF4 includes iso-$C_{17:1}$ I and/or anteiso-$C_{17:1}$ B. Values showed are percentages of total fatty acids with major components highlighted in bold (>5.0%); TR, trace (<1.0%); –, not detected. Fatty acids accounting for <1% in all six strains are not shown.

Supplementary Table S5. General features and genomic and phenotypic characteristics of R1DC41^T strain and closely related members of *Bacillaceae* family. Morphological and physiological data are collected from the relative publications. N/A: no data available; +: positive; −: negative.

**Characteristic**	**R1DC41^T**	**TF-12^T**	**TF-11^T**	**P9^T**	**S6^T**
Isolation source	Mangrove sediments	Sea water	Sea water	Soil	Deep-sea sediment
Cell morphology	Rods	Rods	Rods	Rods	Rods
Colony colour	Pale yellow	Pale orange	Pale yellow	No-pigment	No-pigment
Gram-staining	Variable (−/+)	Variable	+/Variable	+	+
Motility	Non-motile	Flagellum	Flagellum	Flagellum	Motile
Spore formation	+	+	+	+	+
Biotic relationship	Free-living	Free-living	Free-living	Free-living	Free-living
Temperature (°C)					
Range	20–40	10–44	10–47	15–40	10–45
Optimum	20–25	30–37	30–37	30–37	35
NaCl (%)					
Range	1–5	0–18	0–16	0–9	0–8
Optimum	3–4	2–5	2–5	0–1	3
pH (unit)					
Range	6–10	6–8	4.5–9	5.5–10.5	6.0–11.0
Optimum	8.5–9	6–7	6–8	8–8.5	8
Genome size (Mb)	3.2	4.0	4.3	NA	NA
DNA G+C content (mol%)#	38.3	41.7	48.6	39.7	44
Nitrate reduction	−	N/A	N/A	−	−
Oxidase reaction	−	−	−	+	−
Catalase reaction	+	+	+	+	+
Predominant ubiquinone$	MK7	MK7, MK8	MK7, MK6, MK8	MK7, MK6 MK5, MK8	MK-6, MK7
Major cellular fatty acids$	Iso-$C_{15:0}$/ Anteiso-$C_{15:0}$	Iso-$C_{15:0}$/ Anteiso-$C_{15:0}$	Anteiso- $C_{15:0}$/ Iso-$C_{15:0}$	Anteiso- $C_{15:0}$/ Iso-$C_{15:0}$	Iso-$C_{15:0}$/ $C_{14:0}$/ Antesio-$C_{15:0}$
Polar lipids$	DPG, PG, L, AL	DPG, PG, PE, PL, AL	DPG, PG, PE, PL, APL, PL	DPG, PE, PG, PL, AL	DPG, APL, PE, PG, AL

R1DC41^T *Mangrovibacillus cuniculi* (this study); TF-12^T *Rossellomorea aquimaris* [1]; TF-11^T *Rossellomorea marisflavic* [1]; P9^T *Jeotgalibacillus soli* [2]; S6^T *Domibacillus iocasae* [3].
#All G+C content data were obtained from the different genomes.
$Analysis have been performed at DSMZ growing the strains on marine broth, refer to Supplementary Figure S3.

Supplementary Figures

Supplementary Figure S1. (**a**) Representative image of *Avicennia marina* mangrove from which sediments were sampled and (**b**) leaves' litter of mangroves.

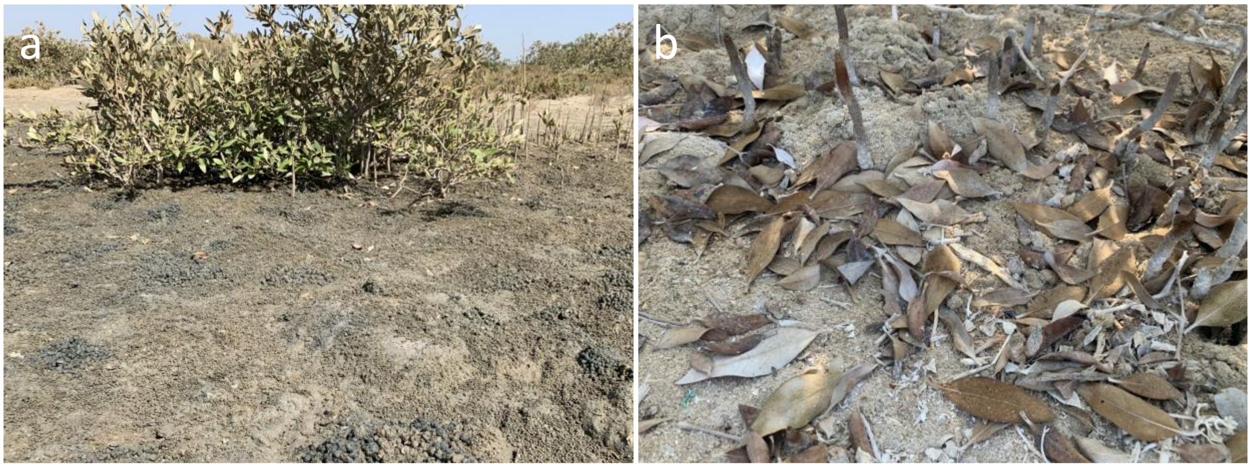

Supplementary Figure S2. (**a**) Comparison of the 16S rRNA gene sequences from the three isolates, namely R1DC41^{T}, R2DC6 and R3DC8. (**b**) ERIC-PCR fingerprinting patterns of the PCR products generated by using the ERIC primers for strains R1DC41^{T}, R2DC6 and R3DC8; ERIC-PCR products are visualized by bioanalyzer Agilent 2100, using high sensitivity dsDNA kit.

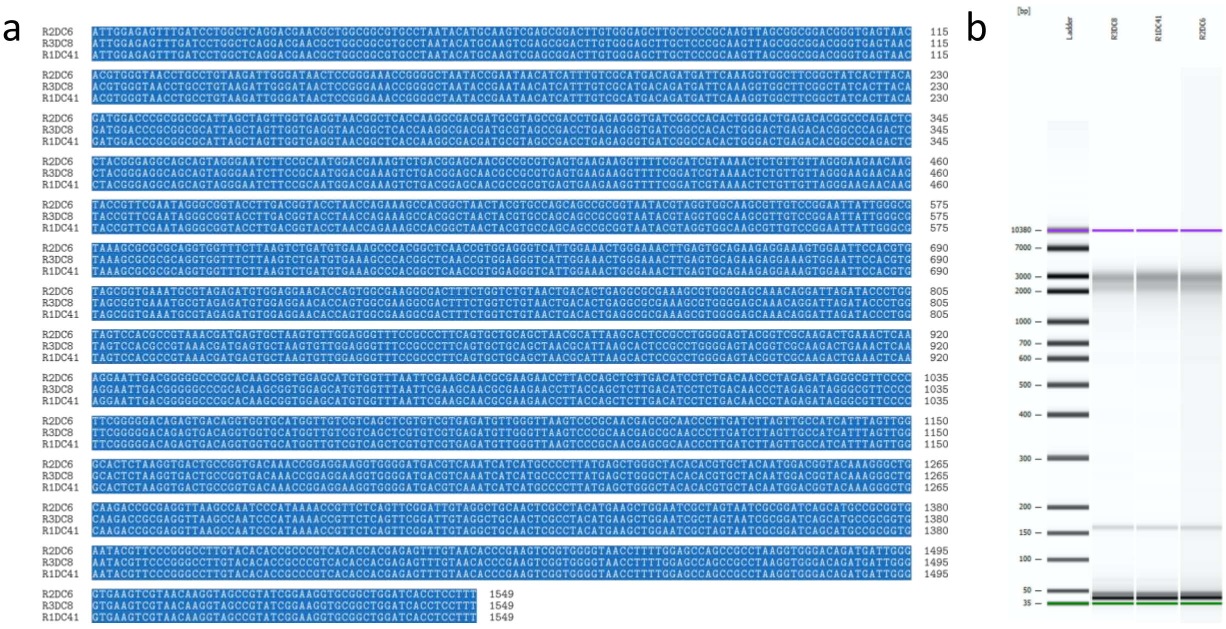

Supplementary Figure S3. *I*dentification *of* polar lipid. Analysis of the five strains were carried out by DSMZ Services, Lebniz-Institut DSMZ. *Mangrovibacillus cuniculi* R1DC41^{T},; *Rossellomorea aquimaris* TF-12^{T} [1]; *Rossellomorea marisflavi* TF-11^{T} [1]; *Jeotgalibacillus soli* P9^{T} [2]; *Domibacillus iocasae* S6^{T} [3].

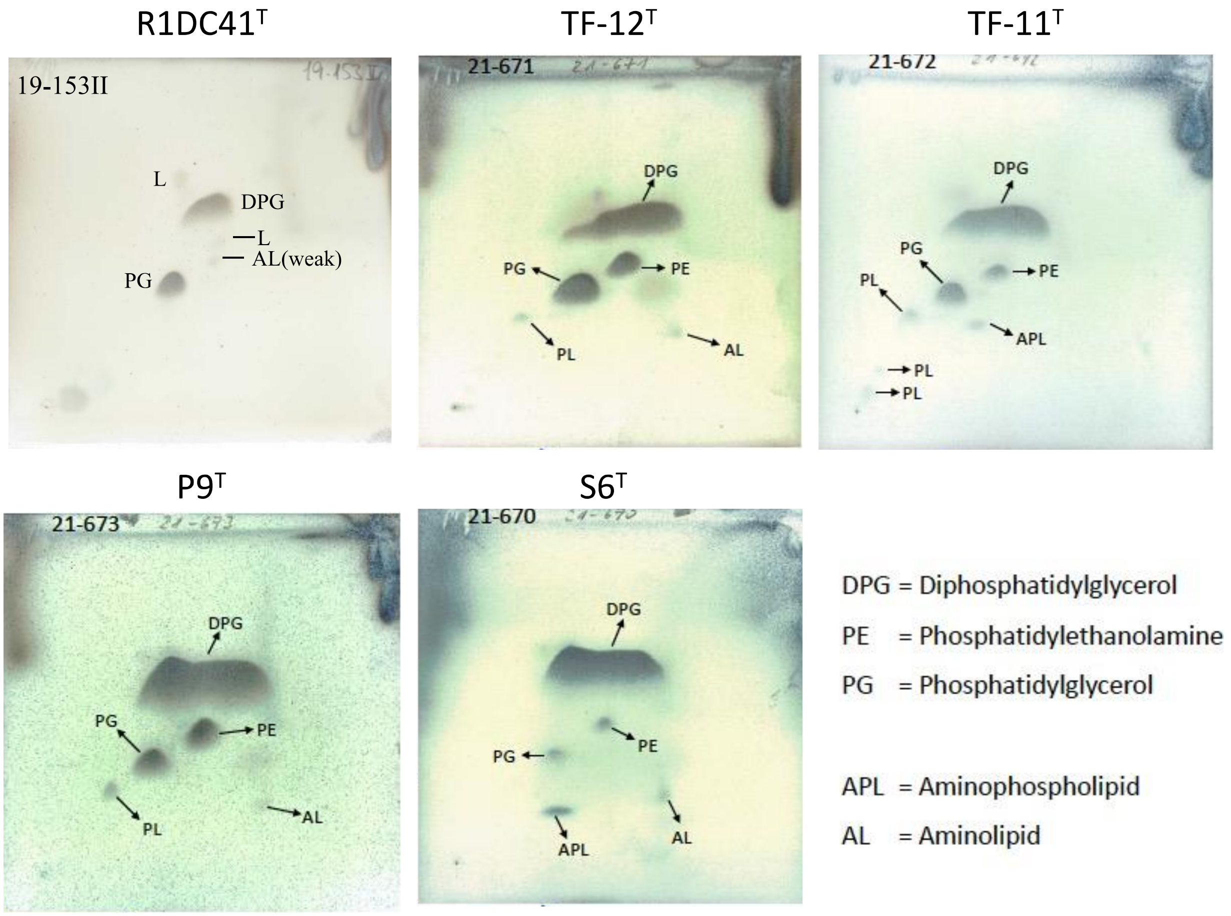

Supplementary Figure S4. (**a**) Graphical circular map of the chromosome and genome features of the R1DC41^{T} strain. From outside to the center: genes on forward strand (blue), genes on reverse strand (green), RNA genes (tRNAs purple, rRNAs blue), GC content, GC skew. The red bar indicates the presence of an integrated phage of 43kb. The predicted *oriC* is also indicated on the map. (**b**) Graphical linear map of the integrated phage in the genome of R1DC41^{T} strain. The purple, black, and orange arrows represent the phage marker proteins, hypothetical proteins, and other CDS, respectively. The red squares represent the attachments sites *attL* and *attR*. The locus tags are indicated at the top of the figure for selected genes.

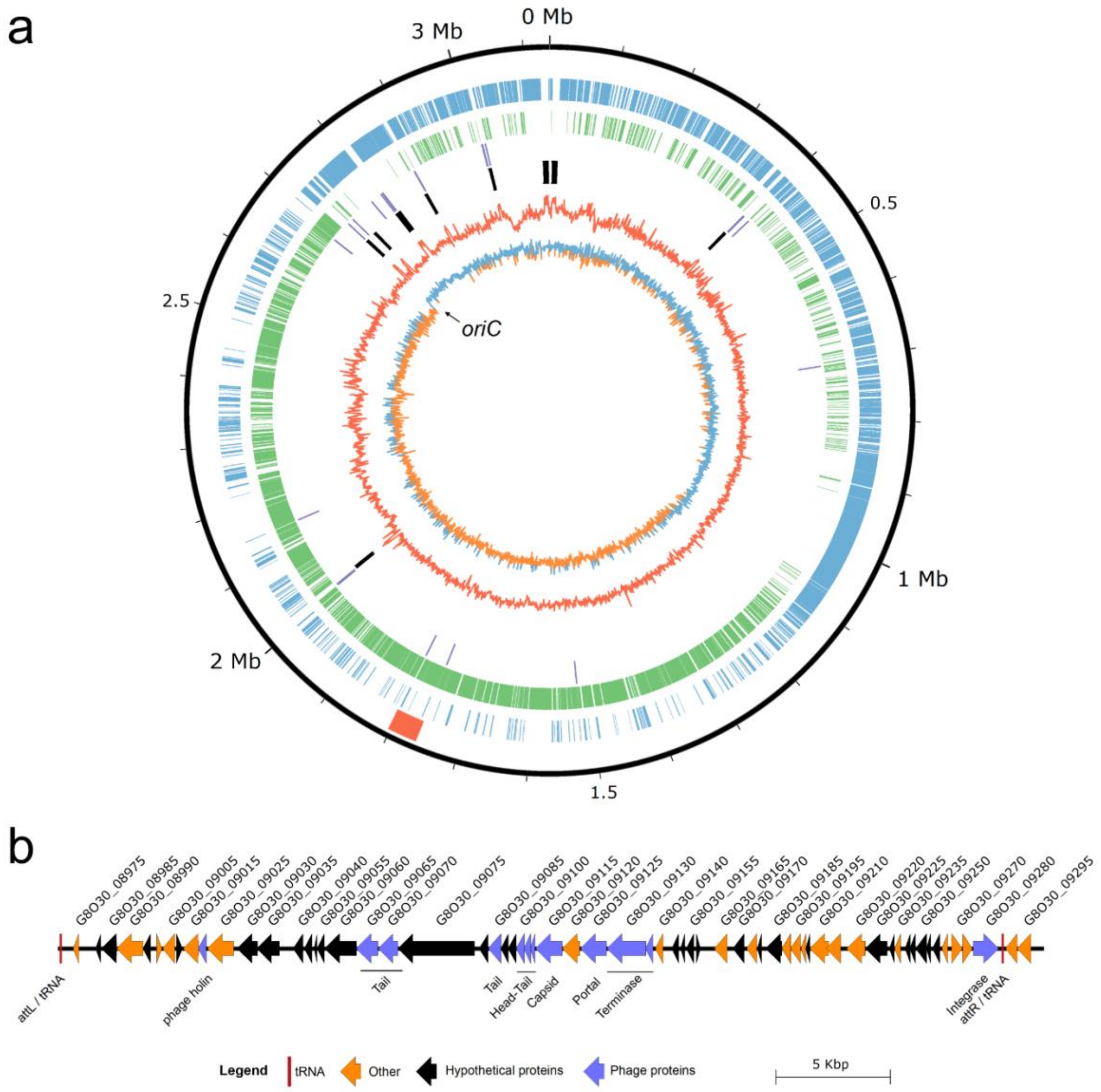

Supplementary Bibliography

1. **Yoon JH, Kim IG, Kang KH, Oh TK, Park YH**. *Bacillus marisflavi* sp. nov. and *Bacillus aquimaris* sp. nov., isolated from sea water of a tidal flat of the Yellow Sea in Korea. *Int J Syst Evol Microbiol* 2003;53:1297–1303.
2. **Cunha S, Tiago I, Paiva G, Nobre F, da Costa MS, *et al.*** *Jeotgalibacillus soli* sp. nov., a Gram-stain-positive bacterium isolated from soil. *Int J Syst Evol Microbiol* 2012;62:608–612.
3. **Sun Q-L, Sun L**. Description of *Domibacillus iocasae* sp. nov., isolated from deep-sea sediment, and emended description of the genus *Domibacillus*. *Int J Syst Evol Microbiol* 2016;66:982–987.
